# Cost-effectiveness analysis of toripalimab plus chemotherapy versus chemotherapy alone for advanced non-small cell lung cancer in China

**DOI:** 10.3389/fimmu.2023.1169752

**Published:** 2023-05-29

**Authors:** Mengdie Zhang, Kai Xu, Yingtao Lin, Chongchong Zhou, Yuwen Bao, Lingli Zhang, Xin Li

**Affiliations:** ^1^ Department of Pharmaceutical Regulatory Science and Pharmacoeconomics, School of Pharmacy, Nanjing Medical University, Nanjing, China; ^2^ Department of Drug Clinical Trial Institution, Fujian Cancer Hospital, Fuzhou, Fujian, China; ^3^ Department of Research Management, Nanjing Stomatological Hospital, Medical School of Nanjing University, Nanjing, China; ^4^ Department of Health Policy, School of Health Policy and Management, Nanjing Medical University, Nanjing, China; ^5^ Center for Global Health, School of Public Health, Nanjing Medical University, Nanjing, China

**Keywords:** toripalimab, PD-1, cost-effectiveness, partitioned survival model, advanced non-small cell lung cancer (advanced NSCLC), chemothearpy, China

## Abstract

**Background:**

Toripalimab is the first domestic anti-tumor programmed death 1 antibody marketed in China. The CHOICE-01 trial (identifier: NCT 03856411) demonstrated that toripalimab plus chemotherapy can significantly improve the clinical outcomes of advanced non-small cell lung cancer (NSCLC) patients. However, whether it is cost-effective remains unknown. Given the high cost of combination therapy, a cost-effectiveness analysis of toripalimab plus chemotherapy (TC) versus chemotherapy alone (PC) for the first-line treatment of patients with advanced NSCLC is required.

**Methods:**

A partitioned survival model was adopted to predict the course of disease in advanced NSCLC patients on TC or PC from the perspective of the Chinese healthcare system over a 10-year horizon. The survival data were obtained from the CHOICE-01 clinical trial. Cost and utility values were obtained from local hospitals and kinds of literature. Based on these parameters, the incremental cost-effectiveness ratio (ICER) of TC vs. PC was measured, and one-way sensitivity analyses, probabilistic sensitivity analyses (PSA), and scenario analyses were performed to assess the robustness of the model.

**Results:**

In the base case, TC was associated with an incremental cost of $18510 and an incremental quality-adjusted life year (QALY) of 0.57 compared with PC, resulting in an ICER of $32237/QALY which was lower than the willingness to pay (WTP) threshold ($37654/QALY), TC was cost-effective. The health utility value of progression-free survival, the price of toripalimab, and the cost of best supportive care were factors that significantly influenced the ICER, but no change in any of them could change the model result. TC showed a 90% probability of being a cost-effective option at a WTP threshold of $37,654/QALY. In the 20 and 30-year time horizons, the results remained unchanged and TC remained cost-effective when the second-line treatment was switched to docetaxel.

**Conclusion:**

At a WTP threshold of $37,654 per QALY, TC was cost-effective compared to PC for patients with advanced NSCLC in China.

## 1 Introduction

People around the world, especially in China, are facing the health issue of lung cancer. According to the latest report published by the Globolan 2020 (1): The incidence and mortality of lung cancer rank first among all malignant tumors in China, with 17.9% and 23.8% respectively, and are both higher than the world average (11.4%, only behind breast cancer (11.7%); 18%). Non-small cell lung cancer (NSCLC) accounts for about 85% of all types of lung cancer (2) and the majority of patients are diagnosed at an advanced stage because of the concealed early symptoms, which results in a dismal prognosis (3). In addition, the burden of disease caused by lung cancer is also considerable. In China, lung cancer accounts for 25.4% of all cancer-related disability-adjusted life years, making it the leading cause of disability-adjusted life year burden (4).

The stage of lung cancer significantly impacts both treatment and prognosis (5). Traditional platinum-based chemotherapy has been the first-line treatment for NSCLC patients (6), but its clinical benefit is modest. The discovery of immune checkpoint inhibitors, represented by programmed death-1(PD-1) and programmed death-ligand 1(PD-L1) antibodies in recent years has opened up new therapy options for advanced NSCLC (7). Numerous clinical trials (8, 9) have shown that chemotherapy plus PD-1/PD-L1 inhibitors can significantly improve the clinical outcomes of patients. Guidelines recommend chemotherapy plus PD-1/PD-L1 inhibitors as the first-line treatment for advanced NSCLC without EGFR or ALK mutations (6, 10). However, high-value antineoplastic medications come at a high cost, thus it is necessary to assess their cost-effectiveness to prevent financial toxicity for patients as a result of high drug prices and to provide the best treatment for patients.

Toripalimab is the first domestic anti-tumor PD-1 antibody marketed in China (11), it is a humanized IgG_4K_ monoclonal antibody special for human PD-1. Clinical studies (12–14) have shown that it has a wide range of anti-tumor activity, including melanoma, lung cancer, gastrointestinal tumors, etc. CHOICE-01 (15) was a randomized, double-blind, placebo-controlled phase III clinical trial conducted in 59 medical centers in China that evaluated the efficacy and safety of toripalimab plus chemotherapy (TC) versus chemotherapy alone (PC) for advanced NSCLC patients. The median follow-up period in this clinical trial was 16.2 months. The findings demonstrated that TC could considerably prolong the progression-free survival (PFS), overall survival (OS), and the anti-tumor response of patients compared to PC, and the toxicity was manageable. The median PFS in the TC group was 2.7 months longer than in the PC group. (8.3 months vs. 5.6 months; HR, 0.49; 95% cl: 0.39-0.61); median OS in the PC group was 17.1 months (95% cl: 14.4 to 22.2) whereas the median OS for the TC arm has not yet reached (95% cl: 21.7 to NE).

Toripalimab for the treatment of melanoma, nasopharyngeal carcinoma, and uroepithelial carcinoma was included in the national reimbursement drug list (NRDL) in 2020 and 2021 (10, 16. 17), respectively. Following the release of its new indication for NSCLC in September 2022 (11), its price may be further reduced after the expiry of the national drug price negotiation (NDPN) on 31 December 2023 (16). However, there is no pharmacoeconomic evaluation of toripalimab for the treatment of advanced NSCLC, so it is necessary to assess the cost-effectiveness of the toripalimab from the perspective of healthcare systems in low-and middle-income countries. In this study, we aimed to evaluate the cost-effectiveness of TC versus PC for the first-line treatment of advanced NSCLC from the perspective of the Chinese healthcare system, to provide evidence for rational drug use in clinical practice and medical decision-making by government departments.

## 2 Data and methods

We conducted a cost-effectiveness analysis based on the CHOICE-01 clinical trial. The Checklist for Economic Evaluation Reporting Standards Statement (CHEERS) was used to guide this article (**Supplementary Material, Table S1**) and details of its methodology are given below.

### 2.1 Population and intervention measure

Patients in this study were assumed to be consistent with those in the clinical trial in that all patients were treatment-naive, locally progressed or metastatic NSCLC, or had finished neoadjuvant/adjuvant therapy ≥ 6 months before enrollment. Non-squamous NSCLC patients with EGFR or ALK driver mutations were disqualified. All enrolled patients were stratified according to baseline characteristics such as PD-L1 expression status, histology (squamous v non-squamous), and smoking status, and were randomly assigned to the study group (TC) and control group (PC) in a 2:1 ratio. The baseline information and subgroups of patients are shown in **Table 1** (15).

**Table 1:** Summary of patient baseline information.

Characteristic	All patients (n=465)		Squamous (n=220)		Non-squamous (n=245)	
	TC (66.5%)	PC (33.5%)	TC (66.8%)	PC (33.2%)	TC (66.1%)	PC (33.9%)
Age, years						
Median age, years(range)	63 (36-75)	61 (29-75)	64 (38-75)	61 (42-75)	62 (36-74)	60 (29-74)
≥65 years	130 (42.1)	55 (35.3)	66 (44.9)	30 (41.1)	64 (39.5)	25 (30.1)
Sex, No. (%)						
Male	247 (79.9)	130 (83.3)	132 (89.8)	66 (90.4)	115 (71.0)	64 (77.1)
ECOG, PS, NO. (%)						
0	66 (21.4)	36 (23.1)	27 (18.4)	16 (21.9)	39 (24.1)	20 (24.1)
1	243 (78.6)	120 (76.9)	120 (81.6)	57 (78.1)	123 (75.9)	63 (75.9)
PD-L1 expression (per IWRS), NO. (%)						
TC≥1%	201 (65.0)	103 (66.0)	96 (65.3)	49 (67.1)	105 (64.8)	54 (65.1)
TC<1%	108 (35.0)	53 (34.0)	51 (34.7)	24 (32.9)	57 (35.2)	29 (34.9)
Smoking status (per IWRS), NO. (%)						
Frequent	213 (68.9)	107 (68.6)	119 (81.0)	59 (80.8)	94 (58.0)	48 (57.8)
Nonsmoker	96 (31.1)	49 (31.4)	28 (19.0)	14 (19.2)	68 (42.0)	35 (42.2)
Clinical stage at study entry, NO. (%)						
IIIB/IIIC	49 (15.9)	23 (14.7)	24 (16.3)	15 (20.5)	25 (15.4)	8 (9.6)
IVA/IVB	260 (84.1)	133 (85.3)	123 (83.7)	58 (79.5)	137 (84.6)	75 (90.4)
Sites of metastases, NO. (%)						
Brain metastases	5 (1.6)	0	3 (2.0)	0	2 (1.2)	0
Hepatic metastases	26 (8.4)	14 (9.0)	16 (10.9)	6 (8.2)	10 (6.2)	8 (9.6)
≥3 sites	53 (17.2)	24 (15.4)	25 (17.0)	8 (11.0)	28 (17.2)	16 (19.3)
Prior therapy, NO. (%)						
Neoadjuvant/adjuvant	14 (4.5)	9 (5.8)	6 (4.1)	2 (2.7)	8 (4.9)	7 (8.4)
Radical surgery	23 (7.4)	15 (9.6)	9 (6.1)	5 (6.8)	14 (8.6)	10 (12.0)
Radiation	11 (3.6)	1 (0.6)	6 (4.1)	0	5 (3.1)	1 (1.2)

TC, toripalimab plus chemotherapy; PC, placebo plus chemotherapy; ECOG, PS, ECOG PS, Eastern Cooperative Oncology Group performance status; IWRS, interactive web response system; PD-L1 expression, programmed death ligand-1.

In 3-week cycles, patients in the TC group received toripalimab 240 mg in combination with standard chemotherapy once per cycle and continued for 4-6 cycles, and then followed by maintenance treatment with toripalimab (± pemetrexed*) until disease progression; patients in the PC arm received placebo once per cycle in combination with standard chemotherapy and continued for 4-6 cycles, followed by maintenance treatment with toripalimab (± pemetrexed*) until disease progression. Patients with disease progression were unblinded and patients from the PC group were allowed to crossover to toripalimab monotherapy, with 13.3% and 65.4% of patients in the TC and PC groups with progressive disease receiving second-line treatment with toripalimab, respectively. The clinical trial made no mention of second-line therapy for the remaining patients, who were assumed to receive the best supportive care based on the recommendations of the guideline (6), patients who did not receive second-line treatment with toripalimab were assumed to receive the best supportive care, with 86.7% and 34.6% of patients with progressive disease in the TC and PC groups, respectively, receiving best supportive care as second-line treatment.

Chemotherapy regimens were based on histological features. Squamous NSCLC patients received paclitaxel plus carboplatin (47.3%) and non-squamous NSCLC patients received pemetrexed plus carboplatin/cisplatin (52.7%) (**Table 2**). In addition, patients with non-squamous NSCLC received toripalimab along with pemetrexed in maintenance therapy*.

**Table 2:** Chemotherapy regimens.

Histology	Therapy	Dosage	Frequency /cycle	Duration	Proportion in TC group	Proportion in PC group
Squamous NSCLC (47.3%)	Paclitaxel	100mg/m^2^	Days 1, 8, 15	4-6	47.6%	46.8%
	Carboplatin	0.4g/m^2^	Day 1	4-6		
Non-squamous NSCLC (52.7%)	Pemetrexed*	500mg/m^2^	Day 1	4-6	42.4%	43.2%
	Cisplatin/ Carboplatin	75mg/m^2^ /0.4g/m^2^	Day 1	4-6		

Pemetrexed*: Maintenance therapy for patients with non-squamous NSCLC was toripalimab plus pemetrexed and for patients with squamous NSCLC was toripalimab alone.

The body surface area (BSA) of patients was set at 1.67 m^2^ (lower limit: 1.4 m^2^, upper limit: 1.94 m^2^) according to a study on individualized administration of NSCLC patients in China (17) and the report on nutrition and chronic disease status of Chinese residents in 2022 (18).

### 2.2 Model overview

**Figure 1:**
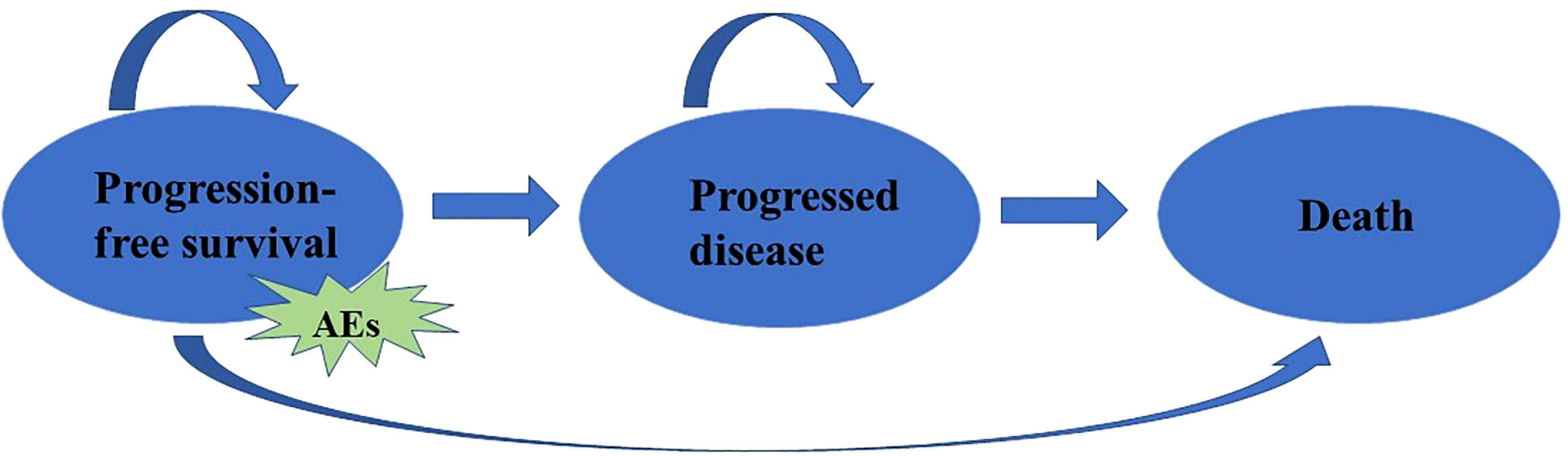
Model structure for advanced NSCLC. AEs: Adverse effects, which were identified by the National Cancer Institute-Common Terminology Criteria for Adverse Events (NCI-CTCAE).

A partitioned survival model was developed in Microsoft Excel^®^ (Microsoft Corp, Redmond, WA, USA) to evaluate the cost-effectiveness of TC versus PC in patients with advanced NSCLC from the perspective of the Chinese healthcare system. In the model, patients were classified into 3 health statuses according to the characteristics of the tumor: progression-free survival (PFS), progressive disease (PD), and death (D). The proportion of patients in different health status at cycle t (3 weeks per cycle) was measured based on the survival curves provided in CHOICE-01 clinical trial. Adverse effects (AEs) were also considered. To simplify the model, we only considered the cost and disutility of grade 3/4 AEs in greater than or equal to 5% of patients in the TC and PC group, the National Cancer Institute-Common Terminology Criteria for Adverse Events (NCI-CTCAE) version 5.0 (19) was used to identify AEs (**Figure 1**).

The proportion of patients in each healthy state at each cycle was calculated as follows:

a) Proportion of patients in PFS was obtained from the PFS curve.
b) Proportion of patients in PD = Proportion of patients who are alive (obtained from OS curve) - Proportion of patients in PFS (obtained from PFS curve).
c) Proportion of patients in D = 1- Proportion of patients who are alive (obtained from OS curve) (20, 21).

GetData Graph Digitizer (version 2.26 www.getdata.graph.digitizer.com) was used to obtain the individual patient data on PFS and OS by taking points from the Kaplan-Meier survival curves reported in clinical trials. And then R software (22) was used to simulate the long-term survival state of patients by selecting the best-fitting parameter distributions to the survival curve from Weibull, Exponential, Gompertz, Gamma, Log-logistic, and Log-normal distributions.

Based on the Akaike Information Criterion (AIC) and Bayesian Information Criterion (BIC), the Log-normal distribution showed the best fit for both PFS and OS in the TC Group and the PC Group. (**Supplementary Material, Table S2, Figure S3**). A half-period correction was used to eliminate discretization-related model bias as the survival curve was divided into monthly periods (23).

The primary outcome was the incremental cost-effectiveness ratio (ICER) which was calculated as the cost per quality-adjusted life year (QALY) gained. The ICER was compared with the preset willingness to pay threshold (WTP) to assess the cost-effectiveness of the two regimens. According to the China Guidelines for Pharmacoeconomic Evaluations (2020) (24), the WTP was set as 3 times China’s per capita GDP in 2021. Sensitivity analyses were performed to evaluate the robustness of the model.

### 2.3 Time horizon and discount rate

Estimates of the number of years of survival for patients with advanced NSCLC were obtained from the National Cancer Institute (NCI) (5), and statistics showed that most patients with advanced NSCLC will die within 10 years (survival rate of 2.7%), so we used a 3-week cycle length and a 10-year lifetime horizon in this study to simulate advanced NSCLC patients’ survival years.

The discount rate for both costs and health outputs was 5% as recommended in the guidelines (24), and sensitivity analysis was performed on a range of 0% to 8%.

### 2.4 Cost and utility

Since our study perspective was the healthcare system, only direct medical costs were considered including drug costs, adverse reaction (ADR) monitoring costs, laboratory test costs, hospitalization, and nursing costs, best supportive care costs, and hospice care costs. Drug costs include chemotherapy drug costs, toripalimab costs, and ADRs treatment costs which were calculated as a one-time cost within the first treatment cycle; laboratory tests aimed at tumor surveillance, which was performed once at baseline, and then every 6 weeks for 12 months and every 9 weeks after 12 months. The costs of best supportive care and hospice care were obtained from a previous study (25), and the other costs were obtained from Fujian Cancer Hospital, which is a tertiary oncology hospital and participated in the CHOICE-01 clinical trial as one of the sub-centers, so the cost information provided by Fujian Cancer Hospital was consistent with the clinical trial and representative. All costs were translated into USD based on the average exchange rate of CNY to USD in 2021 (1 USD=6.4515 CNY) (26) (**Supplementary Material, Table S4**).

The utility values for PFS and PD and the disutility values for ADRs were calculated according to the published literature (25, 27), as patients’ quality of life was not examined in the clinical trials. A utility value of 0 for the death state (**Supplementary Material, Table S4**).

### 2.5 Sensitivity analysis

Several sensitivity analyses were performed to assess the robustness of the model. One-way sensitivity analysis is to analyse the effect of a variable change in a certain range on the results of the model when other variables remain constant. 95% CI is typically used to restrict the range of variance, if there is no 95% Cl, it may vary by ±10% for non-cost parameters and ±20% for cost parameters based on the base case value (**Supplementary Material, Table S4**). In addition, toripalimab, which is within the term of the NRDL agreement, may be subject to renegotiation and reimbursement adjustments after the expiry of the agreement on 31 December 2023 due to its new indication of NSCLC. According to the data provided by the Chinese government website, the average cost reduction of updated drugs in 2019 and 2020 were 26.4% and 14.95%, respectively (28), so to fully consider the impact of toripalimab’s price on the robustness of the model, we assumed a 36% cost variability for toripalimab is 36% (20% up or down on the original float). One-way sensitivity analysis was presented as a tornado diagram.

The probability sensitivity analysis (PSA) was performed by Monte Carlo simulations 1000 times, each of which randomly chose samples from the distributions of the model inputs. Costs were described by gamma distributions, utility values by beta distributions, and BSA by the normal distribution (**Supplementary Material, Table S4**). The PSA was presented by an ICER scatter plot and a cost-effectiveness acceptability curve (CEAC) which reported the probability that TC is more cost-effective at alternative WTP thresholds.

We also conducted scenario analyses of some key assumptions. Firstly, because long-term survival in advanced NSCLC remains uncertain in clinical practice across countries, literature research was conducted and the results showed that the majority of comparable studies in the cost-effectiveness analysis of ICIs for NSCLC were modelled 10 years, 20 years (29), with some model 5 and 30 years of survival (30, 31), but few papers provided a basis for the choice of survival years. In our scenario study, we investigated the effects of 20 and 30 years of time horizon on the model findings to measure the robustness of the model as far as possible. Secondly, as the clinical trial did not mention second-line treatment for patients not treated with toripalimab, we assumed that this population of patients received guideline-based best supportive care, and in the scenario analysis, we analysed the cost-effectiveness of an alternative guideline-recommended second-line treatment: docetaxel, to examine the robustness of the model (6). In summary, we considered 3 scenarios: 20 and 30-year life horizons, and the impact of docetaxel replacing best supportive care as a second-line treatment option for advanced NSCLC on the model outputs. The price of docetaxel was $0.22/mg.

## 3 Results

### 3.1 Base-case analysis

Compared with PC Group, TC Group was associated with an increment cost of $18501 and an increment QALY of 0.57, resulting in an ICER of $32237/QALY (**Table 3**), which was lower than that of the WTP threshold ($37654) (26). TC was cost-effective.

**Table 3:** Baseline results: cost-effectiveness analysis (10 years).

Group	C ($)	E (QALY)	Incr C	Incr E	ICER($/QALY)
PC	26768	0.83	–	–	–
TC	45268	1.40	18501	0.57	32237

TC, toripalimab plus chemotherapy; PC, placebo plus chemotherapy; C, cost; E, effective; Incr C, Incremental Cost; Incr E, Incremental Effectiveness; ICER, Incremental Cost-Effectiveness Ratio.

### 3.2 One-way sensitivity analysis

**Figure 2:**
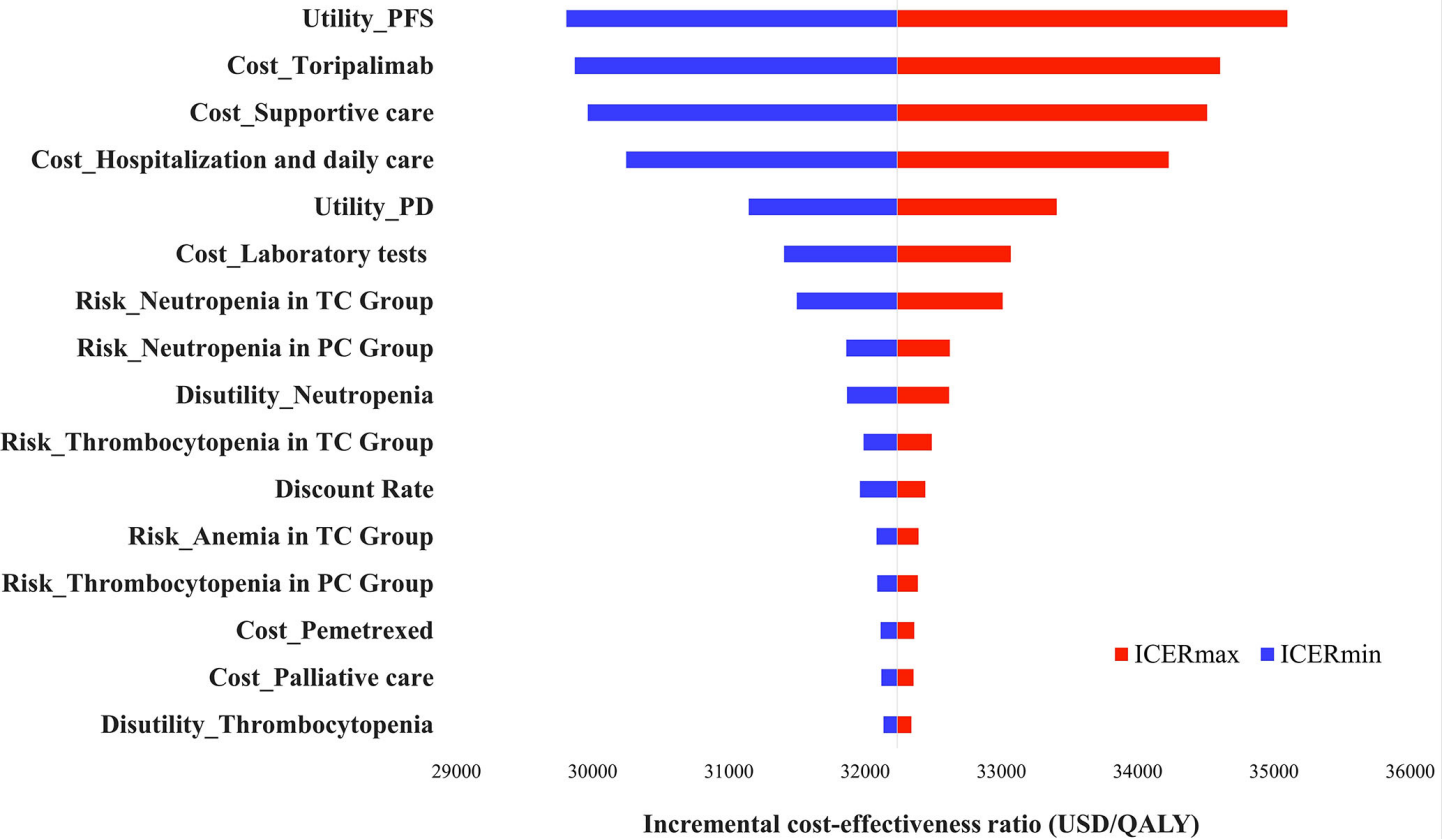
Tornado diagram of one-way sensitivity analyses. TC, toripalimab plus chemotherapy; PC, placebo plus chemotherapy; ICER, Incremental Cost-Effectiveness Ratio.

The one-way sensitivity analyses revealed that the factors that had the greatest impact on the model were the health utility value of PFS, the cost of toripalimab, and the cost of best supportive care, respectively, but no change in any of these parameters could make the ICER higher than the WTP threshold. The model was robust (**Figure 2**).

### 3.3 Probabilistic sensitivity analysis

**Figure 3:**
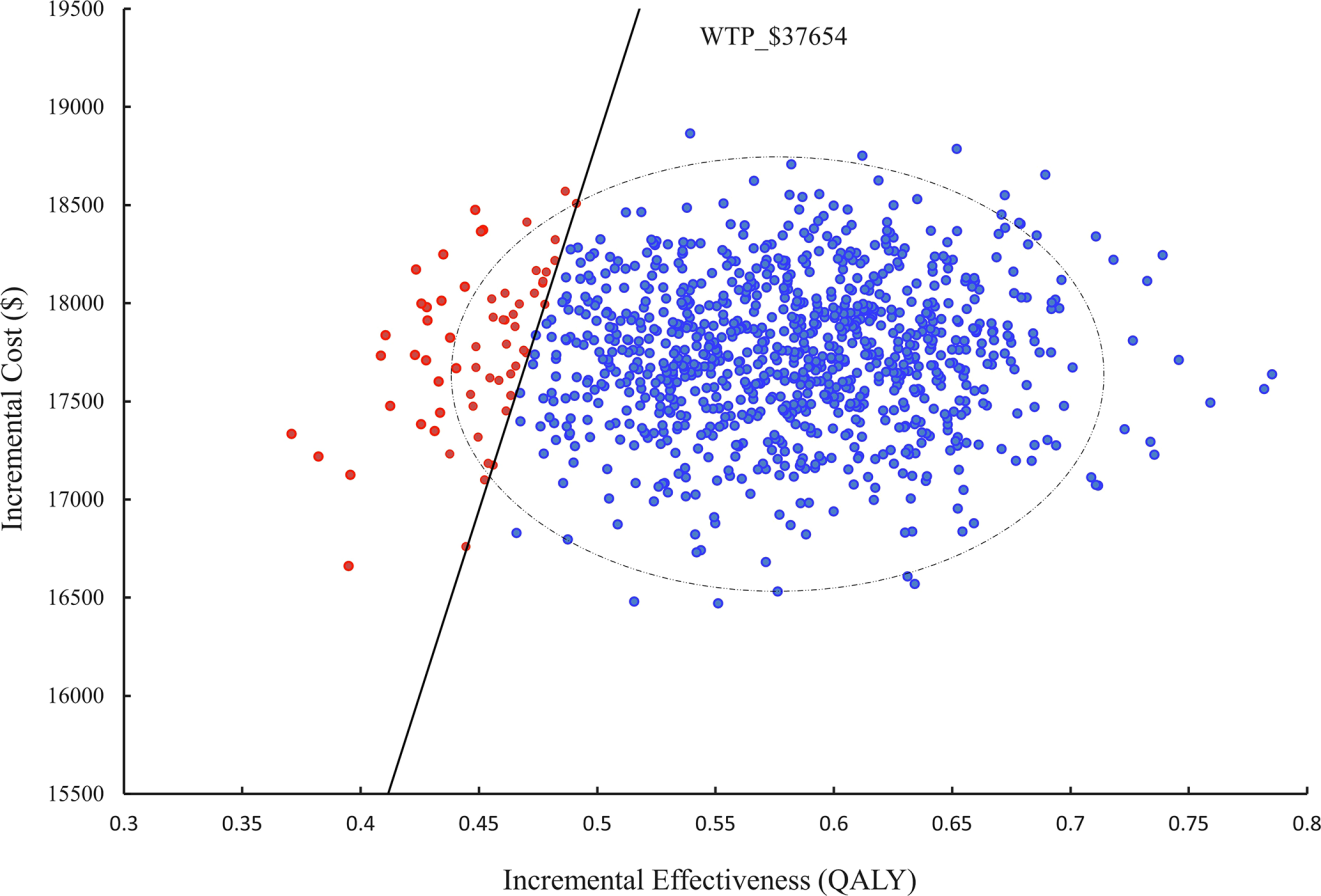
Scatter plot of probability sensitivity analysis. WTP, willingness to pay.

**Figure 4:**
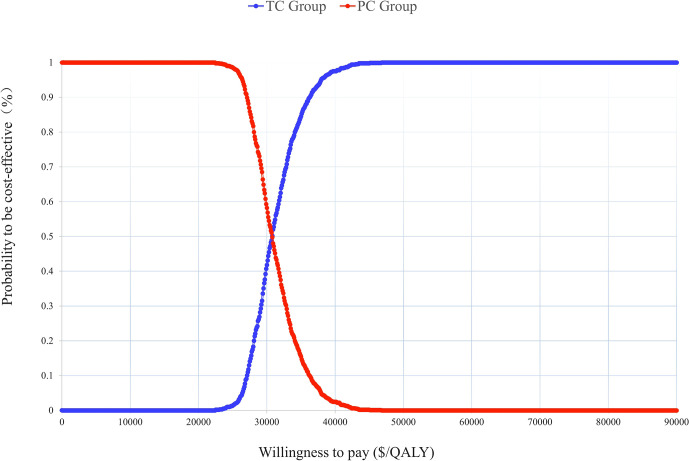
Cost-effectiveness acceptable curve (CEAC). TC, toripalimab plus chemotherapy; PC, placebo plus chemotherapy.

In a probabilistic sensitivity analysis (PSA), 1000 iterations of Monte Carlo simulations were performed and the results were presented as an ICER scatter plot (**Figure 3**) and a CEAC (**Figure 4**), which showed a 90% probability of TC being a cost-effective option at a WTP threshold of $37,654.

### 3.4 Scenario analysis

The results showed that the ICERs were all lower than the WTP threshold at simulated 20,30 years and docetaxel as a second-line treatment ($32494, $32559, $33171 vs. $37654), TC was still cost-effective (**Table 4**).

**Table 4:** Scenario analysis.

Group	C ($)	E (QALY)	Incr C	Incr E	ICER($/QALY)
Scenario 1. Simulated life: 20 years.					
PC	27110	0.83	–	–	–
TC	50190	1.55	23080	0.71	32494
Scenario 2. Simulated life: 30 years.					
PC	27137	0.84	–	–	–
TC	51529	1.58	24393	0.75	32559
Scenario 3. Docetaxel as a second-line treatment option for advanced NSCLC					
PC	35594	0.83	–	–	–
TC	54630	1.40	19037	0.57	33171

TC, toripalimab plus chemotherapy; PC, placebo plus chemotherapy; C, cost; E, effective; Incr C, Incremental Cost; Incr E, Incremental Effectiveness; ICER, Incremental Cost-Effectiveness Ratio; NSCLC, non-small cell lung cancer.

## 4 Discussion

Immune checkpoint inhibitors (ICIs) have drawn a lot of attention since their launch. Although it is a promising field of cancer treatment, it was of considerable significance to the influence of its high cost, especially for low- and middle-income countries. Since 2016, the Chinese government has been implementing an NDPN policy, aiming to reduce the price of innovative medicines with high clinical value (32, 33). High-value innovative medicines, represented by anticancer drugs, have gradually established a value-oriented negotiation access model since 2017 (34), and pharmacoeconomic evaluation has started to play an important role in drug market evaluation. Worth mentioning, innovative drugs that are already in the NRDL do not have to go through negotiations when new medical insurance indications are added, but the drugs also face pressure to reduce prices future (16).

Toripalimab, the first domestic PD-1 antibody with broad antitumor activity marketed in China (11), was approved for marketing by National Medical Products Administration in September 2022 for first-line treatment of advanced non-squamous NSCLC without EGFR/ALK driver mutations. This is the sixth indication for which toripalimab has been approved in China with three indications having previously passed the NDPN and entered the NRDL. In this study, we measured the cost-effectiveness of toripalimab plus chemotherapy for first-line treatment for patients with advanced NSCLC using the price after health insurance reimbursement. The perspective of this research was the Chinese healthcare system, aiming to provide an evidence-based basis for rational clinical use and health insurance decisions. To our knowledge, this is the first analysis to evaluate the cost-effectiveness of toripalimab for advanced NSCLC.

According to our model, TC was cost-effective compared to PC at a WTP threshold of $37,654 per QALY. The utility value of PFS, the price of toripalimab, and the cost of supportive care had the greatest influence on the ICER which were similar to previous studies, but none of them could change the model result. The PSA showed a 90% probability that the ICER was below the WTP threshold. Different survival years and second-line treatment options were also discussed in the scenario analysis and the results remained unchanged, TC was a more cost-effective option for Chinese advanced NSCLC patients.

As a novel treatment option, both imported and domestic ICIs are widely used in China, but the high costs of ICIs can also place a heavy burden on society. Previous studies have demonstrated the cost-effectiveness of imported and domestic ICIs for advanced NSCLC. For imported ICIs, the ICERs for atezolizumab plus chemotherapy (35) and pembrolizumab plus chemotherapy (36) versus chemotherapy alone from the Chinese perspective were $78936/QALY and $107846/QALY, $448414/QALY (pembrolizumab, 2 and 10 mg/kg) showed poor cost-effectiveness. which may be due to the high price of the drugs. A systematic analysis of Pembrolizumab for NSCLC also revealed that it was cost-effective in the US and Switzerland but not in China, France, the UK, or Singapore when combined with chemotherapy (37). For domestic ICIs, the ICERs of sintilimab plus chemotherapy (38) and camrelizumab plus chemotherapy (39) versus chemotherapy alone were $34249/QALY and $63080/QALY, respectively. Compared to other ICIs, toripalimab offers a more cost-effective option with an ICER of $32237/QALY. Additionally, this study was conducted from a Chinese healthcare perspective. Toripalimab combination regimens for the treatment of advanced NSCLC may be more cost-effective from the perspective of the developed country payer. Previously, Junshi Biosciences Co., Ltd has submitted marketing authorization applications to the US FDA, the UK Medicines and Healthcare Products Regulatory Agency, and the European Medicines Agency (11) for two other indications.

Subgroup analyses were not conducted because there are still gaps in the survival data for subgroup participants. Among patients with different PD-L1 expression status and histological features, patients in the PD-L1 tumor cell proportion score ≥50% and non-squamous NSCLC subgroup showed better mPFS and mOS in the clinical trial which may translate into longer QALYs, and resulting in higher ICERs under nearly the same cost of medication. As the clinical experiment moves forward, hopefully, this issue will be looked into more thoroughly.

The study was subjected to several limitations. First, the survival data in this study were obtained from the CHOICE-01 clinical trial rather than real-world data, which may lead to an overestimation of the cost-effectiveness of the combination regimen, as we know that real-world patients’ survival data are typically inferior to those from phase 3 clinical trials which may lead to longer QALYs at the same cost. In addition, as the clinical trials only included Chinese populations, the lack of real-world, global, multi-center survival data and the fact that we discuss the cost-effectiveness of the combination regimen only from the perspective of the Chinese healthcare system may lead to an underestimation of the cost-effectiveness of toripalimab. To our knowledge, China is a developing country, whereas developed countries such as Europe and the US have a much higher GDP per capita and toripalimab may be more cost-effective; Second, the utility and disutility values used in the article were derived from previous studies because there was no information available in the trial on patients’ quality of life. Third, since no second-line treatment regimen was mentioned in the trial, we assumed that all patients received the best supportive care based on guidelines which may not be realistic. Fourth, not all adverse reactions were included in the model. Clinical trials showed a higher incidence of thyroid disease, diarrhea, and edema in the TC group, which may be related to the use of immunosuppressants, but none of the incidences exceeded 5%, so they were not included in the model. In addition, a significant number of patients in the CHOICE-01 trial were switched from the PC group to the TC group after progression due to ethical issues, which may have diluted the survival advantage of the TC group and increased the cost of PC.

Despite these limitations, our study has a high level of confidence. A partitioned survival model was used to simulate the survival of patients with tumors, unlike the markov model, the partitioned survival model does not require the calculation of transfer probabilities between model states, the proportion of patients in each state was directly obtained from the survival curve provided by the clinical trial, avoiding assumptions such as memorylessness and natural mortality and allowing for more accurate modeling of disease events (21). The simulation results showed that the mPFS and mOS of the simulated survival curves were within the 95% confidence interval. In addition, the utility values were derived from previous studies on the quality of life in Chinese NSCLC patients. Last but not least, the incidence and cost of ADRs were analyzed in sensitivity analyses, which showed that these factors had little effect on the model results.

## 5 Conclusions

In conclusion, TC was cost-effective compared to PC for patients with advanced NSCLC at a WTP threshold of $37,654 per QALY in China.

## Data availability statement

The original contributions presented in the study are included in the article/**Supplementary Material**. Further inquiries can be directed to the corresponding authors.

## Author contributions

MZ contributed to data analysis and writing; KX, MZ and YB contributed to modelling and design this study. YL collected the research data. CZ performed the statistical analysis. LZ and XL contributed to manuscript revision, read, and approved the submitted version. All authors contributed to the article and approved the submitted version.
