# Supplementary material for: Cost-effectiveness analysis of toripalimab plus chemotherapy versus chemotherapy alone for advanced non-small cell lung cancer in China
Source: Front Immunol. 2023 May 29;14:1169752. doi: 10.3389/fimmu.2023.1169752 (PMC10258326; doi:10.3389/fimmu.2023.1169752)
Supplement: Supplementary file 1 [file DataSheet_1.docx]

Supplementary Material

Cost-effectiveness analysis of toripalimab plus chemotherapy versus chemotherapy alone for advanced non-small cell lung cancer (NSCLC) in China

Mengdie Zhang^*^, Kai Xu, Yingtao Lin, Chongchong Zhou, Yuwen Bao

*** Correspondence:** Xin Li: xinli@njmu.edu.cn

# Table S1. CHEERS Checklist.

# Table S2. Summary of statistical goodness-of-fit of KM curves in CHOICE-01 trial.

# Figure S3. Best-fitting parametric models for PC Group (A) and TC Group (B).

# Table S4. Base-case values.

# Table S1. CHEERS Checklist.

| **Section/topic** | **Item No** | **Guidance for reporting** | **Reported in Page No.** |
| --- | --- | --- | --- |
| **Title** | | |  |
| Title | 1 | Identify the study as an economic evaluation and specify the interventions being compared. | 1 |
| **Abstract** | | |  |
| Abstract | 2 | Provide a structured summary that highlights context, key methods, results, and alternative analyses. | 1, 2 |
| **Introduction** | | |  |
| Background and objectives | 3 | Give the context for the study, the study question, and its practical relevance for decision making in policy or practice. | 2, 3 |
| **Methods** | | |  |
| Health economic analysis plan | 4 | Indicate whether a health economic analysis plan was developed and where available. | 3 |
| Study population | 5 | Describe characteristics of the study population (such as age range, demographics, socioeconomic, or clinical characteristics). | 3 |
| Setting and location | 6 | Provide relevant contextual information that may influence findings. | 5 |
| Comparators | 7 | Describe the interventions or strategies being compared and why chosen. | 2 |
| Perspective | 8 | State the perspective(s) adopted by the study and why chosen. | 3, 4 |
| Time horizon | 9 | State the time horizon for the study and why appropriate. | 4 |
| Discount rate | 10 | Report the discount rate(s) and reason chosen. | 4 |
| Selection of outcomes | 11 | Describe what outcomes were used as the measure(s) of benefit(s) and harm(s). | 4 |
| Measurement of outcomes | 12 | Describe how outcomes used to capture benefit(s) and harm(s) were measured. | 4 |
| Valuation of outcomes | 13 | Describe the population and methods used to measure and value outcomes. | 3-5 |
| Measurement and valuation of resources and costs | 14 | Describe how costs were valued. | 5 |
| Currency, price date, and conversion | 15 | Report the dates of the estimated resource quantities and unit costs, plus the currency and year of conversion. | 5 |
| Rationale and description of model | 16 | If modelling is used, describe in detail and why used. Report if the model is publicly available and where it can be accessed. | 4 |
| Analytics and assumptions | 17 | Describe any methods for analysing or statistically transforming data, any extrapolation methods, and approaches for validating any model used. | 4 |
| Characterizing heterogeneity | 18 | Describe any methods used for estimating how the results of the study vary for subgroups. | Not applicable |
| Characterizing distributional effects | 19 | Describe how impacts are distributed across different individuals or adjustments made to reflect priority populations. | Not applicable |
| Characterizing uncertainty | 20 | Describe methods to characterise any sources of uncertainty in the analysis. | ___5____ |
| Approach to engagement with patients and others affected by the study | 21 | Describe any approaches to engage patients or service recipients, the general public, communities, or stakeholders (such as clinicians or payers) in the design of the study. | Not applicable |
| **Results** | | |  |
| Study parameters | 22 | Report all analytic inputs (such as values, ranges, references) including uncertainty or distributional assumptions. | Supplementary Material Table 3. Base-case values. |
| Summary of main results | 23 | Report the mean values for the main categories of costs and outcomes of interest and summarise them in the most appropriate overall measure. | Table 2 |
| Effect of uncertainty | 24 | Describe how uncertainty about analytic judgments, inputs, or projections affect findings. Report the effect of choice of discount rate and time horizon, if applicable. | 6 |
| Effect of engagement with patients and others affected by the study | 25 | Report on any difference patient/service recipient, general public, community, or stakeholder involvement made to the approach or findings of the study | Not applicable |
| **Discussion** | | |  |
| Study findings, limitations, generalizability, and current knowledge | 26 | Report key findings, limitations, ethical or equity considerations not captured, and how these could affect patients, policy, or practice. | 7, 8 |
| **Other relevant information** | | | |
| Source of funding | 27 | Describe how the study was funded and any role of the funder in the identification, design, conduct, and reporting of the analysis | 14 |
| Conflicts of interest | 28 | Report authors conflicts of interest according to journal or International Committee of Medical Journal Editors requirements. | 14 |

# *Reference: Husereau D, Drummond M, Augustovski F, et al. Consolidated Health Economic Evaluation Reporting Standards 2022 (CHEERS 2022) Explanation and Elaboration: A report of the ISPOR CHEERS II Good Practices Task Force. Value Health. 2022;25(1):10-31.*

# Table S2. Summary of statistical goodness-of-fit of KM curves in CHOICE-01 trial.

| Table S2 Summary of statistical goodness-of-fit of KM curves in CHOICE-01 clinical trial. | | | | | | |
| --- | --- | --- | --- | --- | --- | --- |
| **Parameters** | **Exponential** | **Weibull** | **Log-normal** | **Log-logistic** | **Gamma** | **Gompertz** |
| **Toripalimab plus Chemotherapy PFS curve** | | | | | | |
| AIC | 1472.656 | 1464.346 | 1422.034^※^ | 1422.973 | 1455.093 | 1473.888 |
| BIC | 1476.389 | 1471.813 | 1429.500^※^ | 1430.440 | 1462.559 | 1481.355 |
| **Chemotherapy alone PFS curve** | | | | | | |
| AIC | 815.0267 | 776.0199 | 762.7184^※^ | 762.9213 | 767.8243 | 797.8497 |
| BIC | 818.0766 | 782.1196 | 768.8182^※^ | 769.0210 | 773.9240 | 803.9494 |
| **Toripalimab plus Chemotherapy OS curve** | | | | | | |
| AIC | 1235.788 | 1235.023 | 1225.401^※^ | 1227.612 | 1233.571 | 1237.634 |
| BIC | 1239.521 | 1242.490 | 1232.867^※^ | 1235.078 | 1241.037 | 1245.010 |
| **Chemotherapy alone OS curve** | | | | | | |
| AIC | 759.7826 | 721.3817 | 714.4752^※^ | 716.8642 | 717.6254 | 737.881 |
| BIC | 763.5159 | 728.8483 | 721.9419^※^ | 724.3309 | 725.0921 | 745.3477 |

^※^, adopted parametric survival function in the model; AIC, Akaike information criterion; BIC, Bayesian Information Criterion; PFS, progression-free survival; OS, overall survival.

# Figure S3. Best-fitting parametric models for survival curves.

# A.


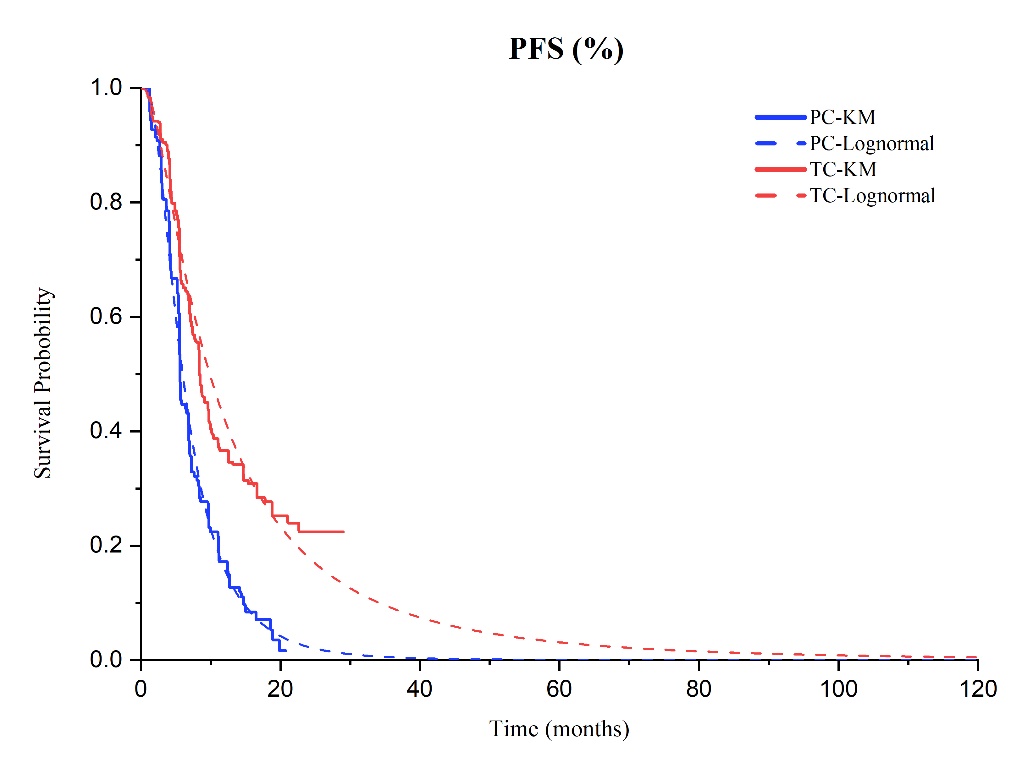


**B.**


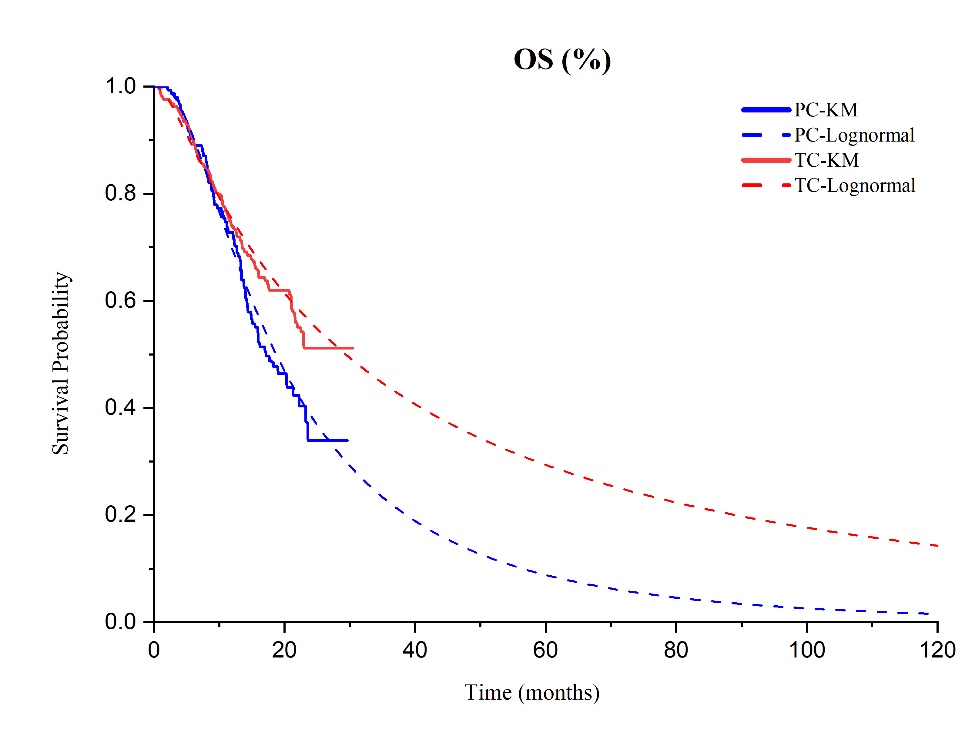


*The replicated Kaplan-Meier survival curves for PC Group (A) and TC Group (B) in the CHOICE-01 trial.*

*PFS, progression-free survival; OS, overall survival; PC, chemotherapy alone; TC, toripalimab plus chemotherapy; KM: Kaplan-Meier survival curves from clinical trial; Lognormal: Log-normal distribution.*

# Table S4. Base-case values.

| **Table S4 Base-case Values** | | | | | |
| --- | --- | --- | --- | --- | --- |
| **Parameter** | **Base** | **Min** | **Max** | **Distribution** | **Source** |
| **Drug costs ($)** |  |  |  |  | Local hospital |
| Toripalimab per cycle | 383.63 | 245.52 | 521.74 | Gamma |  |
| Paclitaxel per cycle | 725.4 | 580.32 | 870.48 | Gamma |  |
| Carboplatin per cycle | 24.61 | 19.69 | 29.53 | Gamma |  |
| Cisplatin per cycle | 15.25 | 12.2 | 18.3 | Gamma |  |
| Pemetrexed per cycle | 67.88 | 54.3 | 81.46 | Gamma |  |
| Docetaxel per cycle | 31.47 | 25.17 | 37.76 | Gamma |  |
| **Tumor evaluation cost ($)** |  |  |  |  | Local hospital |
| Chest CT per time | 99.51 | 79.61 | 119.41 | Gamma |  |
| MRI per time | 159.96 | 127.97 | 191.95 | Gamma |  |
| Bone scanning per time | 99.2 | 79.36 | 119.04 | Gamma |  |
| **Hospitalization and daily care per cycle ($)** | 272 | 217.62 | 326.44 | Gamma | Local hospital |
| **supportive care per cycle ($)** | 338 | 270.4 | 405.6 | Gamma | 24 |
| **palliative care per event（$）** | 2464.5 | 1971.6 | 3696.75 | Gamma | 24 |
| **Adverse Event Treatment Cost（$）** |  |  |  |  | Local hospital |
| Anemia | 145.45 | 116.36 | 174.54 | Gamma |  |
| Neutropenia | 118.54 | 94.83 | 142.25 | Gamma |  |
| Thrombocytopenia | 1052.76 | 842.21 | 1263.31 | Gamma |  |
| **Adverse reaction monitoring cost per cycle （$）** | 14.487 | 11.590 | 17.385 | Gamma | Local hospital |
| **Risk of adverse events in Toripalimab+ chemotherapy group (%)** |  |  |  |  | 15 |
| Anemia | 29.9% | 26.91% | 36.8% | Beta |  |
| Neutropenia | 55.5% | 49.95% | 61.05% | Beta |  |
| Thrombocytopenia | 17.2 | 15.48% | 18.9% | Beta |  |
| **Risk of adverse events in chemotherapy group (%)** |  |  |  |  | 15 |
| Anemia | 35.9% | 32.31% | 39.49% | Beta |  |
| Neutropenia | 53.8% | 48.42% | 59.18% | Beta |  |
| Thrombocytopenia | 17.9% | 16.11% | 19.69% | Beta |  |
| **Health Utility** |  |  |  |  | 26 |
| pfs | 0.804 | 0.7236 | 0.8844 | Beta |  |
| pd | 0.321 | 0.2889 | 0.3531 | Beta |  |
| **Health Disutility** |  |  |  |  | 24 |
| Anemia | -0.073 | -0.0657 | -0.0803 | Beta |  |
| Neutropenia | -0.2 | -0.18 | -0.22 | Beta |  |
| Thrombocytopenia | -0.19 | -0.171 | -0.209 | Beta |  |
| **BSA（m^2^）** | 1.67 | 1.4 | 1.94 | Normal | 18,19 |
| **Discount Rate (%)** | 0.05 | 0 | 0.08 | Fixed | 21 |

PFS: progression-free survival; PD: progression disease; BSA: body surface area(m^2^).
